# Recurrence rate and need for reoperation after surgery with or without internal limiting membrane removal for the treatment of the epiretinal membrane

**DOI:** 10.1186/s40942-017-0101-z

**Published:** 2017-12-11

**Authors:** Fernando José De Novelli, Mauro Goldbaum, Mario Luiz Ribeiro Monteiro, Fabio Bom Aggio, Mario Junqueira Nóbrega, Walter Yukihiko Takahashi

**Affiliations:** 1 Ophthalmology, Universidade da Região de Joinville, Joinville, Brazil; 2 Division of Ophthalmology, University of São Paulo Medical School, São Paulo, Brazil; 3 Rua Camboriu 35, Joinville, SC CEP 89216222 Brazil

**Keywords:** Epiretinal membrane, Internal limiting membrane, Vitrectomy, Metamorphopsia, Optical coherence tomography

## Abstract

**Purpose:**

To compare the recurrence rate and need for reoperation after epiretinal membrane surgery with and without removal of the internal limiting membrane.

**Methods:**

In this retrospective study, 125 patients operated for epiretinal membrane removal were evaluated, with a minimum 6-month follow-up. Removal of the epiretinal membrane (ERM) was performed in 78 patients, while 47 had removal of the epiretinal membrane associated with internal limiting membrane peeling (ERM + ILM).

**Results:**

The mean age in the ERM group was 65.8 years old, ranging from 41 to 80 years old. In the ERM + ILM group, the mean age was 67.2 years old, ranging from 52 to 82 years old. The mean preoperative visual acuity in the ERM group was 20/80p, and in the ERM + ILM group, it was 20/80. The mean postoperative visual acuity in both groups was 20/30. The mean preoperative macular thickness in the ERM group was 467 µm ranging from 281 to 663 µm; in the ERM + ILM group, the preoperative macular thickness was 497 µm, ranging from 172 to 798 µm. After surgery, a reduction in macular thickness was observed in both groups. In the ERM group, the mean macular thickness reduction was 361 ± 101. µm, whereas in the ERM + ILM group, it was 367 ± 75.2 µm. Twenty-two patients presented with a recurrence of epiretinal membrane, of which 16 (20.5%) were from the ERM group and 6 (12.8%) were from the ERM + ILM group (p = 0.39); one patient (2%) was retreated in the ERM + ILM group, whereas 5 patients (6%) where retreated in the ERM group.

**Conclusion:**

We postulate that ILM peeling for the treatment of epiretinal membrane is not a relevant factor either for visual recovery or macular thickness reduction, but it may reduce the recurrence and reoperation rate.

## Background

Epiretinal membrane (ERM) is a retinal condition associated with fibrocellular tissue proliferation on the internal surface of the retina. As the membrane exerts traction force, it generally leads to distorted and worsened vision [1–4].

The level of reduction in visual acuity is variable; however, whenever changes in visual acuity impact individual daily activities, treatment is required. This situation usually occurs with visual acuity below 20/40 or whenever metamorphopsia is highly significant [5, 6].

The treatment of the epiretinal membrane is surgical, through posterior vitrectomy and removal of the ERM with forceps. Some authors believe that if ERM removal is undertaken along with internal limiting membrane peeling, the recurrence rate is lower [7, 8].

The optimal surgical time has not yet been standardized. However, surgery generally occurs when visual acuity is below 20/40. On the other hand, some studies have shown that visual recovery is better with earlier removal of the epiretinal membrane [9–11].

## Methods

In this retrospective study, 125 patients who underwent idiopathic epiretinal membrane surgery were evaluated, with a follow-up time varying from 6 months to 4 years. Surgeries were performed by two surgeons at the Sadalla Amin Ghanem Eye Hospital from 2009 to 2015. One group of patients was operated on, removing the epiretinal membrane (ERM), while another group had the removal of the epiretinal membrane associated with internal limiting membrane peeling (ERM + ILM), based on the surgeon’s assessment and discretion. The groups are similar regarding the mean preoperative best-corrected visual acuity, follow-up time, and the pseudophakic eye proportion during follow-up.

Inclusion criteria were age above 18 years old, visual acuity worse or equal to 0.3 logMAR (20/40), and presenting with a primary epiretinal membrane, as shown by funduscopy and confirmed via optical coherence tomography (OCT).

Exclusion criteria were patients with myopia above 6 diopters, exudative or dry age-related macular degeneration, retinal macular atrophy, diabetic retinopathy of any level of severity, retinal vascular pathology, eyes with previous vitrectomy, a history of uveitis, and less than 6 months follow up. This study was approved by the ethics board of the Sadalla Amin Ghanem Eye Hospital and by the CAPPesq ethics board of research projects from the Sao Paulo University Medical School. This study followed the principles of the Declaration of Helsinki.

Prior to surgery, patients were assessed through a complete eye examination, which included biomicroscopy, applanation tonometry with the Goldmann applanation tonometer, determination of Snellen best-corrected visual acuity with the patient 20 feet (6 m) away using later conversion to logMar to enable statistical analysis, and funduscopy.

Diagnosis of the epiretinal membrane was done clinically through funduscopy and confirmed through optical coherence tomography (OCT); the equipment used was a Stratus HD-OCT for OCT used for examinations performed before 2010, and a Cirrus spectral-domain OCT (Carl Zeiss Meditec) for OCT examinations done after 2010.

Regarding central foveal thickness, which is the distance between the vitreous retinal interface and the inner border of the retinal pigment epithelium and macular volume, we assumed the values calculated by the device in the Macular Cube thickness map. Those eyes presenting with recurrence of the epiretinal membrane were mainly diagnosed through funduscopy and later confirmed by OCT. Repeated surgery in the presence of recurrence of the epiretinal membrane was recommended based on patients’ complaints, as well as on the physician’s clinical assessment.

Transconjunctival posterior vitrectomy 23-gauge was performed utilizing the Accurus 800CS surgical system device (Alcon Surgical, Fort Worth, TX). Phacoemulsification, together with posterior vitrectomy, was performed on those patients with clinically significant cataracts. Detachment of the posterior vitreous was done in cases when it was still attached to the vitreoretinal interface. Upon removal of the vitreous core, the epiretinal membrane was then removed with forceps. In the group of patients where the internal limiting membrane was peeled, the IML was dyed with Brilliant Blue after the steps above and then it was removed from the macular region using the same forceps.

Statistical analyses with *SWILK: Shapiro–Wilk W Test for Normal Data* were used to test the normality of the samples. Measurements of improved visual acuity and macular thickness were compared with the Mann–Whitney test. Recurrence and repeat operation rates were assessed by the Pearson/Yates Chi squared test. This is an exploratory analysis and no sample size calculation was performed. Values of p < 0.05 were considered statistically significant.

## Results

Records of patients diagnosed with epiretinal membrane and operated on by surgeons FJN and MJN at the Sadalla Amin Ghanem Eye Hospital from 2009 to 2015 were retrospectively evaluated. A total of 270 eye records were reviewed, of which 125 were included in the study since they met criteria previously set. Seventy-eight eyes underwent vitrectomy with removal of the epiretinal membrane (ERM group), while 47 eyes underwent posterior vitrectomy associated with removal of the epiretinal membrane and internal limiting membrane peeling (ERM + ILM group). The mean age in the ERM group was 65.8, ranging from 41 to 80 years old. In the ERM + ILM group, the mean age was 67.2, ranging from 52 to 82 years old. The ERM group was composed of 46 men and 32 women. The ERM + ILM group was composed of 24 men and 23 women (Table 1).

**Table 1:** Characteristics of the patients undergoing epiretinal membrane surgery

	Group (ERM) n = 78	Group (ERM+ILM) n = 47	p
Age, years, mean (range) (n = 125)	65.8 ± 10.6 (41–80)	67.2 ± 1.3 (43–80)	0.46
Gender (male/female)	46/32	24/23	O.67
Preoperative visual acuity LogMAR	0.61 (0.20–1.30)	0.60 (0.20–1.30)	0.73
Preoperative visual acuity snellen	20/80p	20/80	1.00
Postoperative visual acuity LogMAR	0.20 (0.00–1.0)	0.20 (0.00–0.90)	0.69
Postoperative visual acuity snellen	20/30	20/30	1.00
Preoperative average central retinal thickness mm, mean (range)	467 ± 85.9 (281–663)	497 ± 123.0 (172–798)	0.16
Postoperative average central retinal thickness mm, mean (range)	361 ± 101.1 (171–647)	367 ± 75.2 (219–582)	0.80
Recurrence	16 (20.5%)	6 (12.8%)	0.39
Reoperation	5 (6.4%)	1 (1.85%)	0.88
Pre treatment pseudophakic	20 (25%)	13 (27%)	0.91
Pos treatment pseudophakic	69 (88%)	43 (91%)	0.81
Follow-up time	46 (6–180)	36 (7–120)	0.06

In the ERM + ILM group, there were two cases (4%) of injection of Brilliant blue dye in the subretinal space in the extra foveal region. In the ERM group, one inadvertent touch with the illuminator probe on the neurosensory retina in the macula occurred. Additionally, two patients received laser photocoagulation on the peripheral retina for treatment of retinal tears.

The mean pre-op visual acuity in the ERM group was 0.61 logMar, and in the ERM + ILM group, it was 0.60 logMar. The mean post-op visual acuity in both groups was 0.20 logMar. No significant difference between groups regarding pre and post-op visual acuity was found. Both groups showed significant improvement of visual acuity upon removal of the epiretinal membrane when compared to preoperative visual acuity (p < 0.001). Patients with preoperative visual acuity better than 20/60 were the ones who achieved better postoperative visual acuity in both groups, with no significant differences between them.

The mean preoperative macular thickness in the ERM group was 467 µm, ranging from 281 to 663 µm; in the ERM + ILM group, the preoperative macular thickness was 497 µm, ranging from 172 to 798 µm. No significant difference was found between the groups (p = 0.16).

After surgery, statistically significant macular thickness reduction occurred in both groups (p < 0.001). In the ERM group, the mean reduction was 361 ± 101.1, ranging from 171 to 647 µm. In the ERM + ILM group, the mean reduction was 367 ± 75.2, ranging from 219 to 582 µm. No significant difference was found between the groups (p = 0.80).

In the ERM + ILM group, 20 eyes (25%) were pseudophakic prior to surgery, 33 eyes (42%) underwent combined phacoemulsification and posterior vitrectomy, and 14 eyes (17%) underwent phacoemulsification after posterior vitrectomy. At the end of follow-up, 63 eyes (81%) were pseudophakic.

In the ERM group, 23 eyes (48%) underwent combined phacoemulsification and posterior vitrectomy, 13 eyes (27%) were already pseudophakic before study enrollment and 12 (25%) had cataract surgery after posterior vitrectomy. Thus, at the end of follow-up, 42 eyes (91%) of patients were pseudophakic.

Twenty-two eyes (17.6%) presented with recurrence of the epiretinal membrane, of which 16 (20.5%) were from the ERM group and 6 (12.8%) from the ERM + ILM group. Although we observed a trend towards a higher recurrence rate in the group where the ILM was not peeled, this difference was not statistically significant (p = 0.39).

Recurrence occurred mainly within 6 months after surgery. However, it was noted after 17 months of follow-up in one patient from the ERM + ILM group.

Retreatment was performed in one eye (2%) from the ERM + ILM group and in 5 eyes (6%) from the ERM group. Considering both groups, a total of 6 patients (4,8%) underwent reoperation.

Two patients refused to undergo a repeated surgical procedure, even with visual acuity worsening and ERM recurrence. Reoperation was not recommended for 12 eyes (66%) that had ERM recurrence since they did not present functional changes or complaints (Table 2).

**Table 2:** Comparing the results of different studies in the literature

Study	ILM peeling		no ILM peeling			ILM peeling	no ILM peeling	
	Eyes N	Mean postoperative BCVA (LogMar) mean/SD	Eyes N	Mean postoperative BCVA (LogMar) mean/SD	p	Recurrence rate	Recurrence rate	p
Kwork [6]	25	0.46/0.37	17	0.65/0.32	0.09	0	3	0.41
Shimada [7]	142	0.26/0.28	104	0.3/0.32	0.53	0	17	<0.001
Lee [24]	21	0.2/0.17	19	0.31/0.23	0.39	0	0	1.00
Kang [25]	28	0.23/0.2	23	0.3/0.31	0.32	1	3	0.62
Ahn [1]	40	0.17/0.17	69	0.11/0.12	1.00	3	14	0.03
Ripandelli [17]	30	0.05/0.08	30	0.06/0.11	0.95	0	0	1.00
Novelli et al.	78	0.2/0.24	47	0.2/0.21	0.85	6	16	0.04

## Discussion

There are two therapeutic options for epiretinal membrane surgery: removal of the epiretinal membrane alone or combined with internal limiting membrane peeling. Several studies have shown improvement of vision using both techniques [4, 12, 13]. However, there is no consensus in the literature on which technique offers the best anatomic and functional results. Some studies show that the peeling of the ILM does not influence vision improvement. One of these studies was carried out by Park [14], who evaluated 44 patients retrospectively. In these patients, there was no significant difference in visual outcome between the studied groups. In the study of Ahn [1], both groups showed similar visual acuity improvement, without a significant difference. However, in the group where the ILM was peeled, vision in the first postoperative month was worse when compared to the group where ILM was not peeled. Nevertheless, after the third postoperative month, improvement occurred and the visual gain was similar in both groups. On the other hand, some studies demonstrate that without internal limiting membrane peeling, the recurrence rate of epiretinal membrane increases, which might be due to remaining fragments of the ERM. Since the internal limiting membrane works as a base and bridge for cellular growth, it may enable cellular proliferation and recurrence of the epiretinal membrane. In addition, peeling of the internal limiting membrane after ERM removal may ensure full ERM removal, therefore lowering the recurrence rate of epiretinal membrane [15–19]. In our study, the group where ILM was peeled presented a lower recurrence rate, suggesting that with the removal of the ILM there is a tendency for less chance of recurrence.

Recently, a comparative meta-analysis and literature review of both techniques was published, with a total of 1367 eyes evaluated, and similar to our study, the results showed that in both groups visual acuity recovery, as well as postoperative macular thickness, were similar, but the rate of recurrence in the group where only ERM was removed was significantly higher.

Peeling of the internal limiting membrane might bring the advantage of lowering the recurrence rate. However, it is a delicate procedure that requires the use of a dye and the careful handling of a tissue that is part of the neurosensory retina. This may, therefore, lead to increased surgical risk. Some studies have shown that dye injection might result in migration of the dye into the subretinal space [20]. In our study, this complication occurred in two eyes (4%).

Another aspect related to peeling of the internal limiting membrane is the mechanical surgical trauma that the procedure might cause. Studies have shown that eyes that underwent ILM peeling developed atrophy spots in the paramacular neurosensory layer, the secondary macular hole and changes in the microperimetry [15, 17, 21–23]. In our study, functional results were similar in both groups. Nonetheless, ILM removal may lead to mild retinal mechanical damage, possibly restricted to a subclinical level.

In the recurrence cases that we followed up, repeat surgery was not recommended in 66% of the cases. Visual acuity was not affected in these cases because the central macular region was spared of traction forces. However, there was a significantly higher proportion of eyes requiring reoperation in the group that underwent ERM removal alone, 6%; in the group that underwent combined ILM peeling, only 1% of eyes needed reoperation, suggesting a trend towards lower recurrence and retreatment rates when this technique is performed.

In conclusion, the anatomic and functional results were similar in both groups. ERM recurrence, as well as the reoperation rate, tended to be higher in the group where the ILM was not removed. It is of note that even in cases of membrane recurrence, the need for reoperation was small in both groups.

## Abbreviations

ERM: epiretinal membrane
ILM: internal limiting membrane peeling
OCT: optical coherence tomography
